# Supplementary material for: Household food insecurity, living conditions, and individual sense of security: A cross-sectional survey among Burkina Faso refugees in Ghana
Source: PLoS One. 2025 Jan 16;20(1):e0317418. doi: 10.1371/journal.pone.0317418 (PMC11737705; doi:10.1371/journal.pone.0317418)
Supplement: S1 File — (DOCX) [file pone.0317418.s001.docx]

**Health Needs Survey for Burkina Faso Displaced Persons and Refugees in Ghana**

Q1 **What is your gender?**

- Male
- Female
- Other (please state) ________________________________________________

Q2 **What is your age in years?**

Q3 **What is your marital status?**

- Never married
- Living together but not married
- Married
- Divorced
- Widowed

Q4 **What is your religious affiliation?**

- Not associated with religion
- Choose not to say
- Christian
- Islam
- Traditional
- Other_ specify…

Q5 **What is your level of education?**

- None
- Basic secondary school
- Complete secondary school
- Vocational education
- Incomplete higher education
- Higher education (Batchelor or Master level)
- Doctoral degree

Q6 **How would you describe the type of settlement in which you lived before the invasion?**

- Urban
- Countryside/rural

Q7  **How would you describe the general settlement where you are currently staying?**

- City/town
- Village
- I'm not sure

Q8 **When did you arrive in your current location?**

- Less than 24 hours ago
- 1-7 days ago
- 8-14 days ago
- 2 weeks - 1 month ago
- More than 1 month ago

Q9 **Where are you currently staying?**

- In housing specifically set up to house refugees or internally displaced people
- With somebody I knew before, such as relatives or friends
- With somebody with whom I was put in touch, for example, friends of friends
- With a local person or people who have offered me accommodation
- Nowhere at the moment - I do not know where I will be staying tonight yet
- In privately rented accommodation
- In a hotel or hostel as a paying guest
- Other (please state) ________________________________________________

Q10 **How would you describe the size of your accommodation?**

- I don't have enough space for myself
- I could do with more space for myself
- I am happy with the size of space I have for myself
- I have more than enough space for myself
- I have too much space for myself

Q11 **Have you received any welfare or housing payments to support yourself in your new accommodation since leaving Burkina Faso?**

- Yes
- No

Q12 To what extent do you agree with the following statements:
**Generally, I feel satisfied with my current living conditions**

- Strongly disagree
- Somewhat disagree
- Neither agree nor disagree
- Somewhat agree
- Strongly agree

Q13 **I feel safe in the place where I am currently**

- Strongly disagree
- Somewhat disagree
- Neither agree nor disagree
- Somewhat agree
- Strongly agree

Q14 **Since my arrival to my new location, I have been made to feel welcome by the local community**

- Strongly disagree
- Somewhat disagree
- Neither agree nor disagree
- Somewhat agree
- Strongly agree

Q15 **Since my arrival to my location, my family and I have experienced hostility or anger from other people in the local community**

- Strongly disagree
- Somewhat disagree
- Neither agree nor disagree
- Somewhat agree
- Strongly agree

Q16 **Thinking about your journey so far since leaving Burkina Faso have you reached your final destination?**

- Yes, I am at my final destination
- No, I am likely to move again within a few days
- No, I am likely to move again within a few weeks
- I don't know yet

Display This Question:

If Thinking about your journey so far since leaving Burkina Faso have you reached your final destination? = No, I am likely to move again within a few days

Or Thinking about your journey so far since leaving Burkina Faso have you reached your final destination? = No, I am likely to move again within a few weeks

Q17 **Where do you think your next destination is likely to be?**

- Somewhere else within Ghana
- Return to my Country
- Travel to another Country
- Other (please state) ________________________________________________
- I don't know

Q18 **Is there a specific town/city or area that you think you are likely to move to next?**

- Yes (please state) ________________________________________________
- No
- I don't know

Q19 **Upon evacuating your country, did you leave anybody from your immediate family in Burkina Faso?**

Yes

No

Q20 **Who out of your immediate family is currently still in Burkina Faso?** *(Feel free to select more than one option)*

- My husband
- My wife
- My father
- My mother
- My stepfather
- My stepmother
- My son(s)
- My daughter(s)
- My brother(s)
- My sister(s)
- My grandfather(s)
- My grandmother(s)
- My father-in-law
- My mother-in-law
- My step-son(s)
- My step-daughter(s)
- My son-in-law
- My daughter-in-law
- My grandson(s)
- My granddaughter(s)

End of Block: About You - 3

Start of Block: Others + General health

Q21 **How many family members, acquaintances, or other travel companions are you currently living with?**

- 0
- 1
- 2
- 3
- 4
- 5
- 6
- 7
- 8
- 9
- 10 or more

Q22 **Do you have any children with you under the age of 18 for whom you have caring responsibilities?**

- Yes
- No

Display This Question:

If Do you have any children with you under the age of 18 for whom you have caring responsibilities? = Yes

Q23
*We will ask some health questions about any children you have toward the end of the survey.*
 **How many children under the age of 18 do you have caring responsibilities for?**

- 1
- 2
- 3
- 4
- 5
- 6
- 7
- 8
- 9
- 10 or more

| Page Break |  |
| --- | --- |

Display This Question:

If Do you have any children with you under the age of 18 for whom you have caring responsibilities? = Yes

Q24 **How many children aged 5 years old or under (0-5) do you have caring responsibilities for?**

- None
- 1
- 2
- 3
- 4
- 5
- 6
- 7
- 8
- 9
- 10 or more

Display This Question:

If How many children aged 5 years old or under (0-5) do you have caring responsibilities for? = 1

Q25 **Does the child for whom you are responsible wear nappies?**

- Yes
- No

Display This Question:

If Does the child for whom you are responsible wear nappies? = Yes

Or Does any of the children for whom you are responsible wear nappies? = Yes

Q26 **How would you describe your current supply and access to nappies?**

- I have run out of nappies, and I do not know where to get more
- I have run out of nappies, but I know where I can get more
- I have some nappies left, but I do not know where I can get more
- I have some nappies left, and I know where I can get more
- I am not sure - somebody else is monitoring nappy supply

Display This Question:

If Does the child for whom you are responsible wear nappies? = Yes

Q27 **Does the child for whom you are responsible have nappy rash?**

- Yes, my child has nappy rash
- No

Display This Question:

If Does any of the children for whom you are responsible wear nappies? = Yes

Q28 **Does any of the children for whom you are responsible have nappy rash?**

- Yes, at least one of my children has nappy rash
- No

| Page Break |  |
| --- | --- |

Display This Question:

If How many children aged 5 years old or under (0-5) do you have caring responsibilities for? = 1

Q29 **Does the child for whom you are responsible typically eat commercially produced baby food?**

- Yes
- No

Display This Question:

If How many children aged 5 years old or under (0-5) do you have caring responsibilities for? = 2

Or How many children aged 5 years old or under (0-5) do you have caring responsibilities for? = 3

Or How many children aged 5 years old or under (0-5) do you have caring responsibilities for? = 4

Or How many children aged 5 years old or under (0-5) do you have caring responsibilities for? = 5

Or How many children aged 5 years old or under (0-5) do you have caring responsibilities for? = 6

Or How many children aged 5 years old or under (0-5) do you have caring responsibilities for? = 7

Or How many children aged 5 years old or under (0-5) do you have caring responsibilities for? = 8

Or How many children aged 5 years old or under (0-5) do you have caring responsibilities for? = 9

Or How many children aged 5 years old or under (0-5) do you have caring responsibilities for? = 10 or more

Q30 **Does any of the children for whom you are responsible eat commercially produced baby food?**

- Yes
- No

Display This Question:

If Does the child for whom you are responsible typically eat commercially produced baby food? = Yes

Or Does any of the children for whom you are responsible eat commercially produced baby food? = Yes

Q31 **How would you describe your current supply and access to baby food?**

- I have run out of baby food, and I do not know where to access more
- I have run out of baby food, but I know where I can access more
- I have some baby food left, but I do not know where I can access more
- I have some baby food left, and I know where I can access more
- I'm not sure - somebody else is monitoring baby food supply

| Page Break |  |
| --- | --- |

Q32 **How would you describe your health right now?**

- Very poor
- Poor
- Average
- Good
- Excellent

Q33 **Do you have any chronic illnesses or health problems? (i.e., that have lasted, or are expected to last, for 6 months or more)**

- Yes
- No

Q34 **Do you currently have, or have been diagnosed with, any of the following diseases or conditions within the past 12 months?** *(Please tick any that apply)*

- Allergies, examples include rhinitis, hay fever, eye inflammation, dermatitis, food allergy or other allergy
- Arthritis
- Asthma
- Cancer
- Consistent back pain (i.e. several episodes of pain, or ongoing back pain)
- Consistent neck pain (i.e. several episodes of pain, or ongoing neck pain)
- Depression or other mental health condition
- Diabetes
- Epilepsy
- Heart attack or other heart related/cardiac condition
- High blood pressure (hypertension)
- HIV
- Hypo- or hyperthyroidism
- Kidney problems
- Liver disease, such as cirrhosis
- Psychiatric condition such as dementia or Alzheimer's
- Stroke (also known as cerebral haemorrhage, cerebral thrombosis)
- Tuberculosis
- Urinary incontinence, problems in controlling the bladder
- Other long-term respiratory condition (e.g., chronic bronchitis, chronic obstructive pulmonary disease, emphysema; please state) ________________________________________________
- Other (please state) ________________________________________________

Display This Question:

If Do you currently have, or have been diagnosed with, any of the following diseases or conditions w... = Allergies, examples include rhinitis, hay fever, eye inflammation, dermatitis, food allergy or other allergy

Q36 You selected that you currently have or have been diagnosed with **allergies (e.g., rhinitis, hay fever, eye inflammation, dermatitis, food allergy or other allergies)** within the past 12 months. 

**How would you describe your current supply and access to the necessary medication to control this condition?**

- I do not take any medication for this condition
- I do not have any medication left, and I don't know where to find more
- I do not have any medication left, but I know where to find more
- I have some medication left, and I don't know where to find more
- I have some medication left, but I know where to find more

Q56 **How would you describe the health of your teeth and gums at the moment?**

- Very poor
- Poor
- Average
- Good
- Excellent

Q57 **How would you describe your access to food at the moment?**

- Very poor
- Poor
- Average
- Good
- Excellent

Q57 **How would you describe your access to clean drinking water at the moment?**

- Very poor
- Poor
- Average
- Good
- Excellent

Q59 **How would you describe your access to bathroom and toilet facilities at the moment?**

- Very poor
- Poor
- Average
- Good
- Excellent

| Page Break |  |
| --- | --- |

Q60 **Do you have access to laundry facilities and soap to wash your clothes?**

- No access
- Very little access
- Yes, some access
- Yes, regular access

Q61 **How would you describe your current access to the use of healthcare facilities?**

- I currently do not have access to healthcare facilities, and I don't know where they are in my community
- I know where healthcare facilities are in my community, but I currently do not have access to them
- I currently have access to healthcare facilities, but I don't know where they are in my community
- I know where healthcare facilities are in my community, and I currently have access to them

Q62 **Do you have difficulty walking 500m on level ground without the use of aid, such as a stick or crutch?**

- Yes
- No

| Page Break |  |
| --- | --- |

Display This Question:

If What is your gender? = Female

Q63 **How would you rate your level of supply and access to required sanitary products, such as tampons or sanitary towels?**

- I do not use sanitary products
- I do not have any sanitary products left, and I don't know where to find more
- I do not have any sanitary products left, but I know where to find more
- I have some sanitary products left, but I don't know where to find more
- I have some sanitary products, and I know where to find more

Display This Question:

If What is your gender? = Female

Q64 **Are you pregnant?**

- Yes, I am pregnant
- I am unsure if I am pregnant
- I am definitely not, or very unlikely to be, pregnant

Display This Question:

If Are you pregnant?  = Yes, I am pregnant

Q65 **How many weeks pregnant are you?**

- 0 to 13 weeks (first trimester)
- 14 to 26 weeks (second trimester)
- 27 to 40 weeks (third trimester)
- I don't know

End of Block: Others + General health

Start of Block: Eye health and hearing

Q66 **How would you describe your vision and eye health?**

- Very poor
- Poor
- Average
- Good
- Excellent

Q67 **Do you wear glasses and/or contact lenses?**

- Yes, I wear glasses only
- Yes, I wear contact lenses only
- Yes, I wear a mixture of glasses and contact lenses
- No

Display This Question:

If Do you wear glasses and/or contact lenses? = Yes, I wear glasses only

Or Do you wear glasses and/or contact lenses? = Yes, I wear contact lenses only

Or Do you wear glasses and/or contact lenses? = Yes, I wear a mixture of glasses and contact lenses

Q68 **Even wearing your glasses and contact lenses, how would you rate your vision?**

- Very poor
- Poor
- Average
- Good
- Excellent

Q69 **Do you wear a hearing aid?**

- Yes
- No

Display This Question:

If Do you wear a hearing aid? = Yes

Q70 **How would you rate your level of supply and access to hearing aid batteries?**

- I do not have any hearing aid batteries left, and I don’t know where to find more
- I do not have any hearing aid batteries left, but I know where to find more
- I have some hearing aid batteries left, but I don’t know where to find more
- I have some hearing aid batteries left, and I know where to find more

| Page Break |  |
| --- | --- |

End of Block: Eye health and hearing

Start of Block: Blast injuries

Q71 **Since the disturbances in Burkina Faso have you been injured as a result of a blast or explosion?**

- Yes
- No

Display This Question:

If Since Russia start its invasion of Burkina Faso have you been injured as a result of a blast or explo... = Yes

Q72 **Which part of your body has been injured?**

- Arms
- Back
- Chest or stomach
- Feet
- Head
- Hands
- Legs
- Neck
- Shoulders
- Other (please specify) ________________________________________________

Q73 Over the past 2 weeks, how often have you been bothered by any of the following?
 **Feeling nervous, anxious, or on edge?**

- Not at all
- Several days
- Over half the days
- Nearly every day

Q74 **Not being able to stop or control worrying?**

- Not at all
- Several days
- Over half the days
- Nearly every day

Q75 **Feeling afraid as if something awful might happen?**

- Not at all
- Several days
- Over half the days
- Nearly every day

End of Block: Mental health and wellbeing - 1

Start of Block: Mental health and wellbeing - 2

Q76 Over the past 2 weeks, how often have you been bothered by any of the following?

**Trouble relaxing?**

- Not at all
- Several days
- Over half the days
- Nearly every day

Q77 **Experiencing nightmares?**

- Not at all
- Several days
- Over half the days
- Nearly every day

Q78 **Experiencing flashbacks to recent events?**

- Not at all
- Several days
- Over half the days
- Nearly every day

End of Block: Mental health and wellbeing - 2

Start of Block: Sleep

Q79 **Generally, over the past 2 weeks, how would you rate your sleep quality?**

- Very poor
- Poor
- Moderate
- Good
- Excellent

Q80 **Over the past 2 weeks, how many hours of sleep per night have you had on average?**

- 0
- 1-3 hours
- 4-6 hours
- 7-9 hours
- 10 hours+

Q81 **Which of the following sleeping materials do you have access to?** *(please select all that apply)*

- Bed sheets
- Blankets or duvets
- Pillows
- Sleeping mats
- Sleeping bags
- None of these

Q82 **How would you describe your current designated sleeping space?**

- I sleep in my own designated sleeping space (i.e., by myself or with my partner)
- I sleep in a shared designated sleeping space with other family members and/or acquaintances

Display This Question:

If How would you describe your current designated sleeping space? = I sleep in a shared designated sleeping space with other family members and/or acquaintances

Q83 **Including yourself, approximately how many people sleep in this designated sleeping space?**

- 2
- 3
- 4
- 5
- 6
- 7
- 8
- 9
- 10-14
- 15-19
- 20+

End of Block: Sleep

Start of Block: Vaccinations

Q84 **Have you been vaccinated against flu during the past 6 months?**

- Yes
- No
- Unsure

Q85 **Have you previously had any doses of a measles or MMR (measles, mumps, rubella) vaccine?** (This may have been when you were a child)

- Yes, 2 or more doses
- Yes, 1 dose
- No, I am unvaccinated against measles
- I cannot remember

| Page Break |  |
| --- | --- |

Q86 **How many doses of the COVID-19 vaccine have you received?**

- 3 doses (including booster)
- 2 doses
- 1 dose
- None
- Unsure

Display This Question:

If How many doses of the COVID-19 vaccine have you received? = 1 dose

Or How many doses of the COVID-19 vaccine have you received? = 2 doses

Or How many doses of the COVID-19 vaccine have you received? = 3 doses (including booster)

Q87 **When did you receive your most recent COVID-19 dose?**

- In the last 4 weeks
- Between 4 and 12 weeks ago
- More than 12 weeks ago
- Unsure

Display This Question:

If How many doses of the COVID-19 vaccine have you received? = None

Q88 **Do you wish to be vaccinated against COVID-19?**

- Yes
- No
- I don't know

Display This Question:

If How many doses of the COVID-19 vaccine have you received? = None

Q89 Please rate your level of agreement to the following statement:

**I wish to receive the COVID-19 vaccine**

- Strongly disagree (Definitely not)
- Somewhat disagree (Likely not)
- Undecided (Neither likely nor unlikely)
- Somewhat agree (Likely)
- Strongly agree (Definitely)

Display This Question:

If Do you wish to be vaccinated against COVID-19? = No

Or Do you wish to be vaccinated against COVID-19? = I don't know

Or Please rate your level of agreement to the following statement: I wish to receive the COVID-19 va... = Strongly disagree (Definitely not)

Or Please rate your level of agreement to the following statement: I wish to receive the COVID-19 va... = Somewhat disagree (Likely not)

Or Please rate your level of agreement to the following statement: I wish to receive the COVID-19 va... = Undecided (Neither likely nor unlikely)

Q90 **Please select your reason(s) for your hesitation toward receiving the vaccination when it becomes available to you.** (you may choose more than one option)

- The vaccine will be unsafe or dangerous
- I don't have enough information about the vaccine
- I will experience side effects and get sick from the vaccine
- I don't need it
- I don't need it - I've already had COVID-19
- I have no access to a COVID-19 vaccine
- The vaccine will not work
- COVID-19 is not severe enough to need a vaccine
- I don't trust the government or the service department
- It is simply a way for pharmaceutical firms to make money
- I will be allergic to the vaccine
- Other (please state) ________________________________________________

| Page Break |  |
| --- | --- |

Q91 **Have you ever had a diagnosis of COVID-19?**

- Yes, I was tested and COVID-19 was medically diagnosed
- I think so, but I was not tested and COVID-19 was not medically diagnosed
- Unsure
- No

Q92 **Have you had any kind of respiratory infectious disease in the last 4 weeks?**

- Yes
- No
- Unsure

End of Block: Vaccinations

Start of Block: Skin health

Q93 **Do you have any persistent itching or need to scratch your skin?**

- Yes, I scratch a lot and it is painful
- Yes, I scratch sometimes
- No I do not have any problems with itching or a need to scratch

Display This Question:

If Do you have any persistent itching or need to scratch your skin? = Yes, I scratch a lot and it is painful

Or Do you have any persistent itching or need to scratch your skin? = Yes, I scratch sometimes

Q94 **Have you had any skin conditions recently diagnosed?**

- No skin conditions recently diagnosed
- Scabies
- Impetigo
- Dermatitis
- Other skin condition (please state) ________________________________________________

Display This Question:

If Do you have any persistent itching or need to scratch your skin? = Yes, I scratch a lot and it is painful

Or Do you have any persistent itching or need to scratch your skin? = Yes, I scratch sometimes

Q95 **For itching, what parts of the body are the most itchy or where you need to scratch the most?**

- Arms
- Back
- Buttocks
- Chest or stomach
- Feet
- Groin
- Hands
- Head
- Legs
- Neck

| Page Break |  |
| --- | --- |

Q96 **Have you noticed other people around you (for example in your household or other nearby refugees) also scratching and itching?**

- Yes, several people
- Yes, one or two other people
- No, not noticed any other people with itching or scratching difficulties

Q97 **Do you have any open wounds or sores?**

- Yes
- No

End of Block: Skin health

**Household food security Issues**

| No | Question | Response Options | CODE |
| --- | --- | --- | --- |
| 1 | In the past four weeks, did you  worry that your household  would not have enough food? | 0 = No (skip to Q2)  1=Yes |  |
| 1a | How often did this happen? | 1 = Rarely (once or twice in the past four weeks)  2 = Sometimes (three to ten times in the past four weeks)  3 = Often (more than ten times in the past four weeks) |  |
| 2 | In the past four weeks, were you or any household member not able to eat the kinds of foods you preferred because of a lack of resources? | 0 = No (skip to Q2)  1=Yes |  |
| 2a | How often did this happen? | 1 = Rarely (once or twice in the past four weeks)  2 = Sometimes (three to ten times in the past four weeks)  3 = Often (more than ten times in the past four weeks) |  |
| 3 | In the past four weeks, did you or any household member have to eat a limited variety of foods due to a lack of resources? | 0 = No (skip to Q2)  1=Yes |  |
| 3a | How often did this happen? | 1 = Rarely (once or twice in the past four weeks)  2 = Sometimes (three to ten times in the past four weeks)  3 = Often (more than ten times in the past four weeks) |  |
| 4 | In the past four weeks, did you or any household member have to eat some foods that you really did not want to eat because of a lack of resources | 0 = No (skip to Q2)  1=Yes |  |
| 4a | How often did this happen? | 1 = Rarely (once or twice in the past four weeks)  2 = Sometimes (three to ten times in the past four weeks)  3 = Often (more than ten times in the past four weeks) |  |
| 5 | In the past four weeks, did you or any household member have to eat a smaller meal than you felt you needed because there was not enough food? | 0 = No (skip to Q2)  1=Yes |  |
| 5a | How often did this happen? | 1 = Rarely (once or twice in the past four weeks)  2 = Sometimes (three to ten times in the past four weeks)  3 = Often (more than ten times in the past four weeks) |  |
| 6 | In the past four weeks, did you or any other household member have to eat fewer meals in a day because there was not enough food? | 0 = No (skip to Q2)  1=Yes |  |
| 6a | How often did this happen? | 1 = Rarely (once or twice in the past four weeks)  2 = Sometimes (three to ten times in the past four weeks)  3 = Often (more than ten times in the past four weeks) |  |
| 7 | In the past four weeks, was there ever no food to eat of any kind in your household because of lack of resources to get food? | 0 = No (skip to Q2)  1=Yes |  |
| 7a | How often did this happen? | 1 = Rarely (once or twice in the past four weeks)  2 = Sometimes (three to ten times in the past four weeks)  3 = Often (more than ten times in the past four weeks) |  |
| 8 | In the past four weeks, did you or any household member go to sleep at night hungry because there was not enough food? | 0 = No (skip to Q2)  1=Yes |  |
| 8a | How often did this happen? | 1 = Rarely (once or twice in the past four weeks)  2 = Sometimes (three to ten times in the past four weeks)  3 = Often (more than ten times in the past four weeks) |  |
| 9 | In the past four weeks, did you or any household member go a whole day and night without eating anything because there was not enough food? | 0 = No (skip to Q2)  1=Yes |  |
| 9a | How often did this happen? | 1 = Rarely (once or twice in the past four weeks)  2 = Sometimes (three to ten times in the past four weeks)  3 = Often (more than ten times in the past four weeks) |  |
|  |  |  |  |

Q113 Please rate the extent of your agreement to the following statement:

**I believe I will return home to Burkina Faso eventually**

- Strongly disagree
- Somewhat disagree
- Neither agree nor disagree
- Somewhat agree
- Strongly agree

Q114 **And finally, please describe any health needs of you or your family that have not been covered in this survey (if applicable)**

________________________________________________________________

________________________________________________________________

________________________________________________________________

________________________________________________________________

________________________________________________________________

End of Block: Final questions
