# Supplementary material for: Household food insecurity, living conditions, and individual sense of security: A cross-sectional survey among Burkina Faso refugees in Ghana
Source: PLoS One. 2025 Jan 16;20(1):e0317418. doi: 10.1371/journal.pone.0317418 (PMC11737705; doi:10.1371/journal.pone.0317418)
Supplement: S2 File — (PDF) [file pone.0317418.s002.pdf]

Burkina Faso Food Insecurity Analysis

Ashley Heinson and Jess Boxall

2024-06-05

R Markdown

This is an R Markdown document that shows the code run to create the prevalence of food insecurity scores in our dataset.

```
df<-read.table("./Data_for_Rasch_Modelling.csv", header=TRUE, row.names=1, sep=',')
#Running Rasch model.
#Install and load RM.weights package (if needed to install)
#install.packages("RM.weights")
library(RM.weights)
```

```
## Loading required package: psychotools
```

```
## Warning: package 'psychotools' was built under R version 4.3.3
```

```
## Loading required package: Hmisc
```

```
##
## Attaching package: 'Hmisc'
```

```
## The following objects are masked from 'package:base':
##
##     format.pval, units
```

```
#Extract FIES variables from dataset
XX<-df[,1:8]
head(XX)
```

```
##   FIES1 FIES2 FIES3 FIES4 FIES5 FIES6 FIES7 FIES8
## 1     1     1     1     1     1     1     1     1
## 2     1     1     1     1     1     1     1     1
## 3     1     1     1     1     1     1     1     1
## 4     1     1     1     1     1     0     1     1
## 5     0     0     1     1     0     1     1     1
## 6     1     1     0     0     1     1     1     1
```

```
#Fit unweighted Rasch model (the weights will be set all to 1)
XX_res = RM.w(XX)
#Display the item severities, standard errors, infits and outfits
cbind("Item sev."=XX_res$b, "St.err."=XX_res$se.b,
      "Infit"=XX_res$infit, "Outfit"=XX_res$outfit)
```

```
##        Item sev.   St.err.     Infit    Outfit
## FIES1 -0.1001277 0.2123048 1.1870229 1.3558346
## FIES2 -0.1001258 0.2123047 0.8320338 0.6553574
## FIES3 -1.0137873 0.2871481 1.0414696 1.1599889
## FIES4  0.5533931 0.1812808 1.1861904 1.2564684
## FIES5 -1.3639711 0.3280969 1.0675536 1.3613567
## FIES6  0.5256657 0.1822807 0.9137263 0.8725076
## FIES7  0.1228662 0.1998816 0.7940007 0.7010190
## FIES8  1.3760276 0.1631277 0.9870739 0.9655232
```

```
#Display respondent severities and measurement errors
cbind("Person par."=XX_res$a, "Error"=XX_res$se.a)
```

```
##        Person par.     Error
##  [1,] -2.996000804 1.4901311
##  [2,] -2.190558913 1.1102387
##  [3,] -1.254969066 0.8677810
##  [4,] -0.582915701 0.7841632
##  [5,]  0.007069452 0.7599077
##  [6,]  0.594036184 0.7800823
##  [7,]  1.256867549 0.8613799
##  [8,]  2.180454007 1.1047318
##  [9,]  2.981796434 1.4901311
```

```
#Display Rasch reliability based on observed distribution of cases across raw scores
XX_res$reliab
```

```
## [1] 0.4337129
```

```
#Display Rasch reliability based on equal proportion of cases in each non-extreme raw score (mor
e comparable across datasets)
XX_res$reliab.fl
```

```
## [1] 0.6997808
```

```
#Calculate observed and expected respondent infit distribution:
quantile.seq = c(0,.01,.02,.05,.10,.25,.50,.75,.90,.95, .98,.99,1)
q.infit = XX_res$q.infit
q.infit.theor = XX_res$q.infit.theor
#Display conditional independence matrix
XX_res$res.cor
```

```
##             FIES1       FIES2        FIES3       FIES4        FIES5
## FIES1  1.00000000  0.12271578  0.131969217 -0.14336038  0.080984568
## FIES2  0.12271578  1.00000000  0.131971351 -0.01046575  0.080987232
## FIES3  0.13196922  0.13197135  1.000000000 -0.05772506 -0.040525477
## FIES4 -0.14336038 -0.01046575 -0.057725061  1.00000000 -0.103360141
## FIES5  0.08098457  0.08098723 -0.040525477 -0.10336014  1.000000000
## FIES6 -0.17237290  0.12859780 -0.007913209 -0.17815212 -0.101003106
## FIES7 -0.11101696  0.07515660 -0.058605935  0.06254129 -0.006691812
## FIES8 -0.32040876  0.07961856 -0.232665836 -0.13079491 -0.175639011
##              FIES6        FIES7       FIES8
## FIES1 -0.172372898 -0.111016961 -0.32040876
## FIES2  0.128597796  0.075156598  0.07961856
## FIES3 -0.007913209 -0.058605935 -0.23266584
## FIES4 -0.178152122  0.062541291 -0.13079491
## FIES5 -0.101003106 -0.006691812 -0.17563901
## FIES6  1.000000000  0.193613486  0.31724012
## FIES7  0.193613486  1.000000000  0.35015951
## FIES8  0.317240117  0.350159514  1.00000000
```

```
#Rerun analysis to save outputs to csv file with country name
XX_res = RM.w(XX, country = "country1", write.file = T)
```

```
# Calculating row sums
row_sums <- rowSums(XX)

# Calculating the median, lower and upper quartiles of the row sums
median_row_sums <- median(row_sums)
lower_quartile_row_sums <- quantile(row_sums, 0.25)
upper_quartile_row_sums <- quantile(row_sums, 0.75)

# Calculating mean of each row
mean_row_sums <- mean(row_sums)

# Calculating standard deviation of each row
sd_row_sums <- sd(row_sums)

# Printing the results
print("Median of Row Sums:")
```

```
## [1] "Median of Row Sums:"
```

```
print(median_row_sums)
```

```
## [1] 8
```

```
print("Lower Quartile of Row Sums:")
```

```
## [1] "Lower Quartile of Row Sums:"
```

```
print(lower_quartile_row_sums)
```

```
## 25%
##   7
```

```
print("Upper Quartile of Row Sums:")
```

```
## [1] "Upper Quartile of Row Sums:"
```

```
print(upper_quartile_row_sums)
```

```
## 75%
##   8
```

```
print("Means of Rows:")
```

```
## [1] "Means of Rows:"
```

```
print(mean_row_sums)
```

```
## [1] 7.280561
```

```
print("Standard Deviations of Rows:")
```

```
## [1] "Standard Deviations of Rows:"
```

```
print(sd_row_sums)
```

```
## [1] 1.332872
```

```
### Plotting the raw values
library(ggplot2)

# Example statistics
mean_value <- 7.28
median_value <- 8
lower_quartile <- 7
upper_quartile <- 8
standard_deviation <- 1.33

# Data for the plot
data_below <- data.frame(
  value = c(mean_value, median_value),
  labels = c("Mean", "Median"),
  y_position = c(0.81, 0.88)  # Adjust y positions to avoid overlap
)

data_above <- data.frame(
  value = c(lower_quartile, upper_quartile),
  labels = c("LQ", "UQ"),
  y_position = c(1.17, 1.1)  # Adjust y positions to avoid overlap
)

# Gradient color bar data
color_bar <- data.frame(
  x = seq(0, 8, length.out = 100),
  y = 1,
  color = seq(0, 1, length.out = 100)
)

# Additional data for the axis/scale numbers
axis_numbers <- data.frame(
  x = 0:8,  # The numbers for the scale
  y = 0.94,  # Adjust the y position to place numbers below the plot
  label = as.character(0:8)
)

# Create the plot
ggplot() +
  geom_tile(data = color_bar, aes(x = x, y = y, fill = color), height = 0.09) +
  scale_fill_gradientn(colors = c("green", "yellow", "orange", "red"), guide = FALSE) +
  geom_text(data = data_below, aes(x = value, y = y_position, label = paste(value, "\n", label
s)), size = 4) +  # Adjust y position for labels
  geom_text(data = data_above, aes(x = value, y = y_position, label = paste(labels, "\n", valu
e)), size = 4) +  # Adjust y position for labels
  geom_text(aes(x = mean_value, y = 1.25, label = paste("SD ±", standard_deviation)), size = 4)
+  # Adjust y position for SD label

  # Add axis numbers below the plot
  geom_text(data = axis_numbers, aes(x = x, y = y, label = label), size = 4) +

  theme_minimal() +
```

```
  theme(
    plot.background = element_rect(fill = "white", color = NA),  # Set background to white
    axis.title.x = element_blank(),
    axis.text.x = element_blank(),
    axis.ticks.x = element_blank(),
    axis.title.y = element_blank(),
    axis.text.y = element_blank(),
    axis.ticks.y = element_blank(),
    panel.grid = element_blank(),
    plot.title = element_text(hjust = 0.5)  # Center the title
  ) +
  coord_cartesian(ylim = c(0.7, 1.4)) +  # Fix the y-axis limits
  ggtitle("Summary Statistics Visualization")  # Add title
```

```
## Warning: The `guide` argument in `scale_*()` cannot be `FALSE`. This was deprecated in
## ggplot2 3.3.4.
## ℹ Please use "none" instead.
## This warning is displayed once every 8 hours.
## Call `lifecycle::last_lifecycle_warnings()` to see where this warning was
## generated.
```

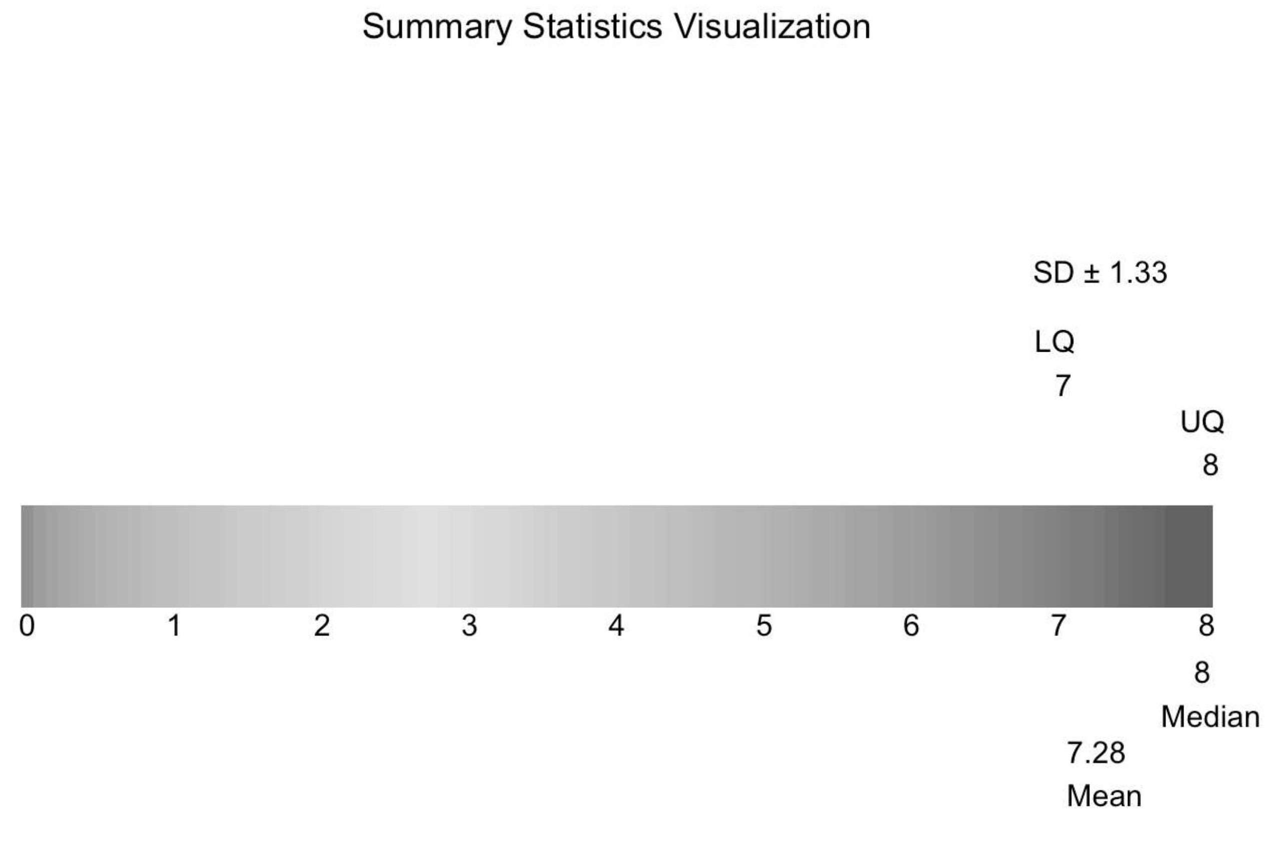


```
# Save the plot
#ggsave("./Results/gradient_plot.png")
#ggsave("./Results/gradient_plot.png", width = 8, units = "in")
```

Post R Analysis

To equate data to global standards, this output analysed manually using the FAO Excel template. The protocol recommended this method and provided up-to-date Gallup World Poll data (2020-2022) 1.

1. Viviani S. Manual for the implementation of the FAO Voices of the Hungry methods to estimate food insecurity: RM.weights packae in R. Rome: Food and Agriculture Organisation, 2016.
