# Supplementary material for: Household food insecurity, living conditions, and individual sense of security: A cross-sectional survey among Burkina Faso refugees in Ghana
Source: PLoS One. 2025 Jan 16;20(1):e0317418. doi: 10.1371/journal.pone.0317418 (PMC11737705; doi:10.1371/journal.pone.0317418)
Supplement: S3 File — (PDF) [file pone.0317418.s003.pdf]

UNIVERSITY FOR DEVELOPMENT STUDIES

Tel: 03720-93382/26634/22078
Email: registrar@uds.edu.gh
Website: www.uds.edu.gh

P. O. Box TL 1350
Tamale, Ghana

Our Ref: UDS/RB/112/22

Your Ref:..

OFFICE OF THE REGISTRAR

Date: 12TH OCTOBER, 2022.

PROF. SHAMSU DEEN ZIBLIM,
INUSAH ABDUL-WAHAB,
AHMED ISSAHAKU TAHIDU AND
MICHAEL HEAD(PhD)

ETHICAL APPROVAL NOTIFICATION

With reference to your request for ethical clearance on the research proposal titled **"Health Needs Survey for Burkina Faso Displaced Persons and Asylum Seekers in Ghana"** I write to inform you that the University for Development Studies Institutional Review Board (UDSIRB) found your proposal including the consent forms to be satisfactory and have duly approved same. The mandatory period for the approval is six (6) months, starting from 12th October, 2022 to 12th March, 2023.

Subject to this approval, you are please required to observe the following conditions:

1. That the anonymity of the respondents shall be guaranteed as mentioned in the consent forms.
2. That you will acknowledge the source of the data collected in any publication related to this research.
3. That you will submit a field report and a copy of the research report to the UDSIRB.
4. That you may apply to the UDSIRB for any amendments relating to recruiting methods, informed consent procedures, study design and research personnel.
5. That you will strictly abide by the code of conduct of this University.

Please do not hesitate to refer any issue (s) that you may deem necessary for the attention of the Board.

Thank you.

Prof. Nafiu Amidu
Member, UDSIRB
For: Chairman, *UDSIRB*
Cc: file
